# Enzyme and Isoenzyme Alterations in Friend Disease

**DOI:** 10.1038/bjc.1970.43

**Published:** 1970-06

**Authors:** D. M. Turner, P. J. Dawson

## Abstract

**Images:**


### ENZYME AND ISOENZYME ALTERATIONS IN FRIEND DISEASE

D. M. TURNER* AND P. J. DAWSON

*From the Departments of Clinical Biochemistry and Pathology, Royal Victoria Infirmary, Newcastle-upon-Tyne*

Received for publication February 11, 1970

**SUMMARY.—The activities of certain enzymes in the tissus of mice infected with Friend virus have been studied. Progressive increases in LDH, G6PDH and 6PGDH have been observed in the spleen and liver concomitant with malignant transformation. Plasma LDH activity was also observed to increase over the period of study, but changes in the isoenzyme distribution probably resulted from contamination of the Friend virus preparation with lactic dehydrogenase virus. The tissue enzyme changes are similar to those observed in human malignancies.**

IT is well known that alterations in the activity of enzymes occur in a variety of animal and human malignant tissues (Douglas, 1963). Moreover, a considerable amount of information exists regarding lactate dehydrogenase (LDH) isoenzyme changes in cancer (Pfleiderer and Wachsmuth, 1961; Goldman, Kaplan and Hall, 1964; Saito, Ohira and Kanamaru, 1968). These are characterised by a relative increase in the proportions of the electrophoretically slowest moving zones, LDH 4 and LDH 5. Infection of cells in culture with Adenovirus 12, which produces malignant transformation, is associated also with a similar change in LDH isoenzyme distribution (Latner, Gardner, Turner and Brown, 1964).

Increase in pentose phosphate cycle activity (van Vals, Bosch and Emmelot, 1956) and increases in the activities of two pentose phosphate cycle enzymes, glucose-6-phosphate dehydrogenase (G6PDH) and 6-phosphogluconate dehydrogenase (6PGDH) (Latner, 1964), have also been associated with malignant change.

A progressive rise in plasma LDH activity has been observed in mice bearing a variety of transplantable tumours (Hseih, Suntzeff and Cowdry, 1955) as well as in mice with certain viral infections (Adams, Rowson and Salaman, 1961; Wenner, Millian, Mirand and Grace, 1962; Mahy, Rowson and Salaman, 1964). The interpretation of enzyme assays in such mice has to take into account possible contamination by lactic dehydrogenase virus (Riley, Lilly, Huerto and Bardell, 1960). This agent, which has now been found in association with a great many transplantable tumours and virus preparations (see review by Notkins, 1965), causes a rise in the activity of several serum enzymes, including LDH, and has a synergistic effect on glycolysis in tumour-bearing animals (Riley, 1963*a*, *b*).

The experiments to be described were designed to determine if the abovementioned biochemical changes associated with malignant transformation occurred during infection of mice with Friend virus, which produces a malignant reticulum cell proliferation in the spleen (Friend, 1957).

* Present address: Department of Pharmacology, Tobacco Research Council Laboratories, Harrogate.

### MATERIALS AND METHODS

BALB/c mice and Scott-Russ rats were obtained from our own colony. The origin of our strain of Friend virus and the methods employed in the preparation of virus pools have been described (Fieldsteel, Dawson and Bostick, 1961). Friend virus from the 8th passage in BALB/c mice was used in the first two experiments. Because the results indicated that this preparation of Friend virus, like virtually all others, contained lactic dehydrogenase virus, a strain of virus that had been passaged in rats was used for the third experiment.

Thymic tissue from 4 rats inoculated when newborn with Friend virus (Dawson, Rose and Fieldsteel, 1966) and killed 155–209 days later with advanced lymphatic leukaemia was made into a pool. Six of 20 young adult BALB/c mice inoculated i.p. with 0·25 ml. of this material developed typical Friend disease which was confirmed histologically. Splenic tissue from five of these killed 89 days post inoculation was made into a pool and inoculated into a further group of 10 young adult BALB/c mice, all of whom rapidly developed Friend disease (mean spleen weight 35 days post inoculation was 2·0 g.). Their spleens were passed once more in BALB/c mice in the same manner. The spleens from two of the latter with typical Friend disease were used as a source of virus in our third experiment. This virus will be referred to as Friend virus (RP).

In each experiment, 18 young adult BALB/c mice were inoculated i.p. with approximately $10^{2\cdot7}$ $ID_{50}$ of either Friend virus or Friend virus (RP). Normal control mice were killed at the beginning of each experiment and test animals were killed 7, 14, and 21 days after inoculation. Each mouse was anaesthetised with intraperitoneal Nembutal and exsanguinated by cardiac puncture using heparin as anticoagulant. Plasma and red cells were separated by centrifugation. Haemolysed plasma was discarded. Spleens and livers were removed and after a part of the spleen had been fixed for histological examination they were stored at —40° C. When splenic stem cells were assayed these were obtained before the tissue was frozen.

Infected tissues and normal tissues for comparison were finely chopped while frozen, and the mince suspended in ice cold physiological saline. This suspension was homogenised in a Griffith's glass tube homogeniser and a portion of homogenate was retained for nitrogen determination. The homogenate was then centrifuged at 100,000 *g*, using a Spinco preparative ultracentrifuge. The supernatant was removed and the pellet resuspended in saline. After recentrifugation, the washings were added to the original supernatant and adjusted to fixed volume. The extracts were then dispensed into small tubes for storage. Samples to be assayed for G6PDH and 6PGDH were stored at —40° C. if they could not be assayed immediately. Those to be assayed for LDH were stored at the same temperature after fortification of the solution with neutralised nicotinamide adenine dinucleotide at a concentration of 10 mg./ml.

Total nitrogen was determined by Natelson's (1961) micro-Kjeldahl technique except that 0·005 M sulphuric acid was used for titration of the ammonia in boric acid, and 5 mg. copper sulphate was employed as the catalyst. The volume of homogenate digested was 0·1–0·3 ml., depending on the approximate tissue concentration. A reagent blank, a standard ammonium sulphate solution (1 mg. nitrogen/ml.) and a serum of known nitrogen content (Versatol, Warner-Chilcott Labs, New Jersey, U.S.A.) were routinely assayed each time.

A stem cell preparation was made from fresh mouse spleen by pressing a crude homogenate in cold phosphate-buffered saline through a 60 mesh wire gauze and the resultant suspension centrifuged at 150 ***g*** for 5 minutes. The pellet was resuspended in ice cold phosphate-buffered saline to a volume of 2 ml. Preferential lysis of red cells was carried out by adding 6 ml. of cold distilled water quickly and, after agitating for 1 minute, 2 ml. of 3·5% aqueous sodium chloride were added to restore isotonicity. The haemolysate was centrifuged for 5 minutes at 150 ***g*** to yield a residue of stem cells with a deep red supernatant. The latter was removed and the cells washed twice with cold physiological saline. Microscopic examination of the final preparation showed that the stem cells were intact. The cells were then resuspended in water and disrupted by freezing and thawing. Supernatant fluid was retained for enzyme assay after ultracentrifugation.

Red cells were washed three times with ice cold saline. An equal volume of cold water was then added to the cells and the suspension frozen and thawed. The lysate was centrifuged at 100,000 ***g*** for 30 minutes and the supernatant removed for enzyme assay.

Lactate dehydrogenase activity was assayed using the spectrophotometric method of Bergmeyer, Bernt and Hess (1963). Glucose-6-phosphate dehydrogenase and 6-phosphogluconate dehydrogenase activities were assayed by a modification of the method of Glock and McLean (1953). The optimal pH for the enzymes from mouse tissues was found to be 9·0 for G6PDH and 7·7 for 6PGDH; otherwise the method was as reported by these authors. Results were expressed as standard international units at 25° C. and for this purpose an experimentally determined temperature coefficient of 1·6 for the range 20–30° C. was employed.

All enzyme activities were corrected to standard international units at 25° C. and expressed in terms of nitrogen content of the homogenate. Results were analysed statistically using Student's " t " distribution.

A sample of each tissue supernatant and plasma was subjected to vertical electrophoresis on starch gel (Latner, 1967). Electrophoresis was performed at 4° C. in a boric acid/sodium hydroxide buffer system (Smithies, 1955) with a voltage gradient of 6 volts/cm. It was allowed to proceed for 2 hours. The LDH isoenzyme activity of a slice of the gel was displayed by the method of Latner and Skillen (1961). Enzyme activities representing G6PDH and 6PGDH were also visualised on other slices using the following incubation mixture: 0·3 M Tris-HCl buffer pH 7·6 (13 ml.); M $MgCl_2$ (1 ml.); 0·025 M glucose-6-phosphate, disodium salt (1 ml.) or 0·025 M 6-phosphogluconate trisodium salt (1 ml.); nicotinamide adenine dinucleotide phosphate (5 mg.); 3-(4,5-dimethyl thiazolyl-2)2,5-diphenyl tetrazolium bromide (4 mg.) and phenazine ethosulphate (0·25 mg.). Gel slices were incubated at 37° C. in the dark for 30 minutes. A quantitative assay was performed on the LDH isoenzymes of plasma and stem cells using reflectance densitometry (Latner and Turner, 1967).

### RESULTS

A preliminary study of the isoenzyme distributions in a variety of mouse tissues was undertaken and the results are illustrated in Fig. 1. All tissues tested contained 5 LDH isoenzymes with a similar distribution to that reported by other investigators (Plagemann, Gregory, Swim and Chan, 1963; Warnock, 1964). LDH 5 moved anodally and had a slightly different mobility in each tissue extract. Addition of serum proteins to the extracts resulted in a uniform mobility of LDH 5.

Liver, spleen, and red cells contained predominantly one zone of activity, namely LDH 5. In this respect mouse red cells differ markedly from their human equivalents which contain predominantly LDH 1 and LDH 2.

In the first experiment the mean spleen weights at the end of the first, second and third weeks after the inoculation of Friend virus were 230, 630, and 1210 mg. respectively (normal mean spleen weight = 110 mg.). Histological examination revealed the presence of proliferating reticulum cells typical of Friend disease. In some spleens at the end of the first week the disease was limited to 10–20 small foci; in others about 60% of the spleen was involved. By the end of the third week the splenic pulp was completely replaced by reticulum cells. Spleens of the same

TABLE I.—*Enzyme Activities in Mouse Plasma and Tissues During Friend Virus Infection Expressed as Standard International Units per mg. Homogenate Nitrogen ± Standard Deviation*

| | Enzyme | Control | Day 7 | Day 14 | Day 21 |
|---|---|---|---|---|---|
| | | | *Experiment* 1 | | |
| Spleen | LDH | 3·10 ±0·32 | 5·28 ±0·36[a] | 6·74 ±1·19[a] | 5·40 ±1·58[e] |
| | 6PGDH | 0·039±0·002 | 0·060±0·014[c] | 0·072±0·011[a] | 0·067±0·003[a] |
| | G6PDH | 0·065±0·003 | 0·192±0·055[a] | 0·254±0·050[a] | 0·314±0·065[a] |
| Liver | LDH | 6·91 ±0·54 | 9·30 ±2·18[d] | 9·53 ±1·48[c] | 8·51 ±1·45[d] |
| | 6PGDH | 0·029±0·002 | 0·028±0·005 | 0·037±0·008[d] | 0·032±0·005 |
| | G6PDH | 0·012±0·001 | 0·020±0·002[a] | 0·041±0·014[a] | 0·056±0·009[a] |
| Red Cells | LDH | 1·28 ±0·22 | 1·33 ±0·16 | 1·71 ±0·35[d] | 1·81 ±0·35[b] |
| | 6PGDH | 0·015±0·001 | 0·016±0·003 | 0·015±0·002 | 0·017±0·002 |
| | G6PDH | 0·023±0·003 | 0·024±0·002 | 0·024±0·004 | 0·025±0·003 |
| Stem Cells | LDH | 0·44 | 4·35 | 5·60 | 1·60 |
| | 6PGDH | 0·06 | 0·07 | 0·16 | 0·15 |
| | G6PDH | 0·05 | 0·13 | 0·53 | 0·59 |
| Plasma | LDH | 0·78 ±0·26 | 2·73 ±0·63[a] | 5·14 ±1·00[a] | 5·88 ±1·50[a] |
| | | | *Experiment* 2 | | |
| Spleen | LDH | 3·40 ±0·92 | 4·19 ±1·62 | 6·01 ±1·31[b] | 3·16 ±0·59 |
| | 6PGDH | 0·038±0·006 | 0·061±0·011[b] | 0·062±0·017[c] | 0·066±0·020[c] |
| | G6PDH | 0·064±0·004 | 0·106±0·026[b] | 0·134±0·043[b] | 0·152±0·047[b] |
| Liver | LDH | 6·28 ±0·82 | 8·17 ±0·78[b] | 8·50 ±1·62[e] | 5·50 ±0·38 |
| | 6PGDH | 0·030±0·004 | 0·028±0·004 | 0·026±0·003 | 0·028±0·004 |
| | G6PDH | 0·013±0·004 | 0·016±0·005 | 0·020±0·002[b] | 0·024±0·002[a] |
| Plasma | LDH | 0·78 ±0·26 | 1·69 ±0·51[b] | 2·77 ±0·31[a] | 2·94 ±0·72[a] |
| | | | *Experiment* 3* | | |
| Spleen | LDH | 3·10 ±0·32 | 6·29 ±2·05[b] | 7·45 ±0·93[a] | 6·47 ±1·28[a] |
| | 6PGDH | 0·039±0·002 | 0·049±0·008[c] | 0·056±0·009[b] | 0·065±0·014[b] |
| | G6PDH | 0·065±0·003 | 0·117±0·024[a] | 0·155±0·033[a] | 0·166±0·053[a] |
| Liver | LDH | 6·19 ±0·54 | 7·46 ±1·71 | 9·83 ±1·87[a] | 9·23 ±1·42[a] |
| | 6PGDH | 0·029±0·002 | 0·027±0·001 | 0·028±0·002 | 0·028±0·001 |
| | G6PDH | 0·012±0·002 | 0·022±0·003[a] | 0·032±0·006[a] | 0·033±0·005[a] |
| Plasma | LDH | 0·77 ±0·26 | 1·34 ±0·23[a] | 1·52 ±0·43[b] | 1·58 ±0·48[b] |

* Infected with Friend virus (RP).
[a] $P < 0·0005$.
[b] $P < 0·0025$.
[c] $P < 0·005$.
[d] $P < 0·025$.
[e] $P < 0·01$.

---

EXPLANATION OF PLATE

FIG. 1.—LDH isoenzyme patterns in normal tissues of the mouse.

Turner and Dawson.

order of magnitude with comparable histological changes were observed in subsequent experiments.

The results of the enzyme determinations in the first experiment are shown in Table I. There was a significant increase in plasma LDH activity over the period of study, levels being significantly elevated by the first week. Fig. 2 shows the plasma LDH isoenzyme patterns as drawn by the densitometer. A relative increase in LDH 5 occurred which was quite marked by the third week of infection. Due to overlap of the zones of LDH activity it was not possible to quantitate individual isoenzymes.

The disease produced statistically significant increases in the LDH activity of spleen during the first 2 weeks of infection, but LDH activity fell by the third week. Levels of 6PGDH and G6PDH were also significantly elevated by the first week and increased over the next 2 weeks.

FIG. 2.—Densitometric scans of serum LDH isoenzymes from the first experiment. The numerals to the right of each scan denote the time in weeks after Friend virus infection.

Changes in the levels of liver enzymes were somewhat similar to those in spleen. The activity of G6PDH rose significantly and 6PGDH activity showed a small but significant increase by the second week. As with spleen, the LDH activity, after rising for the first 2 weeks of the disease, dropped sharply by the third week. Red cell enzyme activities did not differ significantly from normal during the first week of infection. However, a small but significant increase occurred in LDH activity by the second week which was maintained in the third week.

The stem cell preparations showed similar changes to whole spleen. With regard to LDH isoenzymes, there were no observable differences in distribution in crude liver and spleen homogenates and red cells, but stem cells showed progressive changes throughout the 3 weeks of study. Fig. 3 shows the isoenzyme distributions obtained by the densitometric scanning. As with plasma isoenzymes the separation of LDH 4 and LDH 5 was not complete. Nevertheless it was possible to observe a relative increase in LDH 4 over the 3 weeks of study. No changes in the enzymes 6PGDH and G6PDH (as visualised after starch gel electrophoresis) were observed in any tissues, each enzyme migrating as one zone of activity.

In a second experiment, spleen and liver supernatant fractions, as well as plasma, were examined with results essentially similar to the first experiment (Table I). The increases in enzyme activity, though still significantly elevated above the normal range, were in some cases not quite so marked. The activity of G6PDH in liver increased significantly, though not as markedly as in the first experiment, and 6PGDH activity was unchanged. Again, however, the decrease of LDH activity in spleen and liver by the third week after infection was observed. Plasma LDH levels showed a significant but less marked rise over the period of study, but the relative shift in LDH isoenzymes observed in the initial experiment still occurred.

The Friend virus used in the two experiments described almost certainly contained lactic dehydrogenase virus. To obviate this, Friend virus (RP), which had been passaged once in rats, was used. The course and histological appearances of Friend disease induced by this strain were similar to that of our standard mouse-passaged strain.

FIG. 3.—Densitometric scans of splenic stem cell LDH isoenzymes from the first experiment. The numerals to the right of each scan denote the time in weeks after Friend virus infection.

The results (Table I) show that increases in the three enzymes assayed occurred as in the previous two experiments, though they were of a smaller order than those in experiment 2. Though the plasma LDH activity rose by a factor of 2, which was statistically significant in relation to the control level, no changes in isoenzyme distribution were observed during the 3 weeks of infection. Statistical analysis of plasma LDH activities in the three experiments showed that the maximum levels attained in the Friend virus (RP) experiment were significantly lower than the corresponding levels in the first two experiments ($P < 0{\cdot}0025$).

### DISCUSSION

All the tissues examined were found to contain no more than five isoenzymes, the distributions being in good agreement with those reported by Plagemann *et al.* (1963) and Warnock (1964). Though tissues such as heart and kidney showed LDH isoenzyme distributions similar to their human equivalents, one notable difference was observed in the red cells. The mouse red cell pattern was characterised by one zone of activity, LDH 5, which is in marked contrast to the isoenzyme pattern of human erythrocytes. Since a number of mouse tumours contain only this isoenzyme (unpublished observations) as do tissues such as skeletal muscle and liver, it would appear that this isoenzyme has a similar metabolic function in regard to anaerobiosis to its human equivalent (Dawson, Goodfriend and Kaplan, 1964). An attempt has been made to correlate the presence of LDH 5 with the presence of a nucleus as in the primitive red cell (Vessell, 1963), since immature human red cells and normal duck red cells contain both a nucleus and a large proportion of LDH 5 activity. This argument would not hold for the mouse red cell.

The tissues chosen for examination during the course of Friend virus infection, namely liver, spleen and red cells, contained predominantly one isoenzyme, LDH 5, and no differences in isoenzyme distribution resulted from Friend virus infection, as might have been expected by comparison with malignant change in human tissue. The progressive increase in the relative proportions of LDH 4 in the stem cells is similar to the change observed in the livers of rats bearing dimethyl benzanthracene-induced tumours (Kline and Clayton, 1964). Plasma LDH isoenzymes, however, were altered in such a way as to produce a relative increase of LDH 5, a finding which had been seen previously in the plasma of tumour-bearing mice infected with lactic dehydrogenase virus (Plagemann *et al.*, 1963). This virus contaminates virtually all preparations of Friend virus. The changes observed in plasma LDH in the first two experiments are similar to those induced by the former virus, suggesting that our preparation of Friend virus is no exception. To what extent then can the changes observed be attributed to Friend virus? Enzyme activities of LDH and G6PDH in the livers and stem cell preparations from the spleens were significantly elevated in all three experiments. 6PGDH was significantly elevated in the spleens of all infected animals by the second week, but only in the first experiment was there a small rise in liver 6PGDH. The latter could represent early involvement of the liver by Friend disease, as disease developed rather more quickly in this experiment.

Lactic dehydrogenase virus does not survive in the rat for more than a week or two (Notkins, Berry, Moloney and Greenfield, 1962; Plagemann *et al.*, 1963). This observation has been utilised to free preparations of Moloney and Friend viruses from lactic dehydrogenase virus (Notkins *et al.*, 1962; Mahy *et al.*, 1964). In our third experiment the Friend virus used had been in rats for between 155 and 209 days and passaged subsequently in a very small number of mice from a colony apparently free from lactic dehydrogenase virus. This strain of virus (Friend virus [RP]) produced comparable changes in the spleen and liver enzymes to the other preparations of Friend virus and a two-fold increase in plasma LDH levels. Characteristically, lactic dehydrogenase virus affects only the plasma enzymes and neither LDH in the spleen and liver nor G6PDH in serum, spleen, and liver are affected (Plagemann, Watanabe and Swim, 1962; Bailey, Stearman and Clough, 1963; Warnock, 1964). For these reasons it seems probable that the enzyme changes in the spleens and livers of mice infected with Friend virus were due to this virus *per se* rather than contaminating lactic dehydrogenase virus.

In very careful experiments, Mahy *et al.* (1964) reported that no plasma enzyme increases whatsoever were observed during the first 10 days of Friend virus infection, although in mice with advanced disease a moderate increase in LDH was observed. They did not, however, determine the LDH levels in the spleen and liver. In comparing studies on Friend disease in different laboratories, allowance must be made for the fact that Friend virus is a mixture of several different agents. In addition to lactic dehydrogenase virus these include what Mirand has termed focus-forming virus, a lymphatic leukaemia-inducing virus, and a virus-inducing polycythaemia (Mirand, Steeves, Avila and Grace, 1968). These components are probably present in different proportions in various strains; for example, the BALB/c derived strain used in our laboratory is free from the polycythaemia-inducing component (Mirand *et al.*, 1968).

An increase in pentose phosphate shunt activity reflecting an increased nucleic acid synthesis has been observed in normal tissues (Beaconsfield and Reading, 1964) as well as in malignant tissues (Latner, 1964; Thiery and Willighagen, 1965). LDH activity of liver, spleen and stem cells, which rose significantly during the first 2 weeks, dropped sharply on the third week. Since it has been reported (Crispens, 1963) that serum LDH rises terminally in mice with chemically induced tumours, then the sharp fall in organ activity by the third week of Friend virus infection might indicate that in the spleen and liver the cells have become permeable as a result of viral damage to such an extent that the LDH activity could leak out. Similar observations of enzyme leakage from virus-damaged cells in culture have been made (Latner and Turner, unpublished observations). However, if such LDH leakage occurred, one might expect the serum isoenzyme pattern to show a relative increase in LDH 5 which was not observed with Friend virus (RP). Therefore, other as yet unknown factors may be involved. The only evidence of change produced by removal of the lactic dehydrogenase virus was a diminution in the rise in plasma LDH. The synergistic effect of lactic dehydrogenase virus on the metabolism of some tumours (Riley, 1963*a*) did not appear to occur in Friend disease.

The authors wish to thank Professor A. L. Latner for advice and helpful discussion, and Mrs. Wendy Rose for technical assistance. These studies were performed while the authors were in receipt of grants from the British Empire Cancer Campaign for Research.

### REFERENCES

Adams, D. H., Rowson, K. E. K. and Salaman, M. H.—(1961) *Br. J. Cancer*, **15**, 860.
Bailey, J. M., Stearman, M. and Clough, J.—(1963) *Proc. Soc. exp. Biol. Med.*, **114**, 148.
Beaconsfield, P. and Reading, H. W.—(1964) *Nature, Lond.*, **202**, 464.
Bergmeyer, H.-U., Bernt, E. and Hess, B.—(1963) in 'Methods of Enzymatic Analysis', edited by Bergmeyer, H.-U. Verlag Chemie. New York (Academic Press), p. 736.
Crispens, C. G., Jr.—(1963) *J. natn. Cancer Inst.*, **30**, 361.
Dawson, D. M., Goodfriend, T. L. and Kaplan, N. O.—(1964) *Science, N.Y.*, **143**, 929.
Dawson, P. J., Rose, W. M. and Fieldsteel, A. H.—(1966) *Br. J. Cancer*, **20**, 114.
Douglas, W. R.—(1963) *Br. J. Cancer*, **17**, 415.
Fieldsteel, A. H., Dawson, P. J. and Bostick, W. L.—(1961) *Proc. Soc. exp. Biol. Med.*, **108**, 826.
Friend, C.—(1957) *J. exp. Med.*, **105**, 307.
Glock, G. E. and McLean, P.—(1953) *Biochem. J.*, **55**, 400.
Goldman, R. D., Kaplan, N. O. and Hall, T. C.—(1964) *Cancer Res.*, **24**, 389.

HSIEH, K.-M., SUNTZEFF, H. AND COWDRY, E. V.—(1955) *Proc. Soc. exp. Biol. Med.*, **89**, 627.
KLINE, E. S. AND CLAYTON, C. C.—(1964) *Proc. Soc. exp. Biol. Med.*, **117**, 891.
LATNER, A. L.—(1964) *Proc. Ass. clin. Biochem.*, **3**, 120.—(1967) in ' Advances in Clinical Chemistry ', edited by Sobotka, H. and Stewart, C. P. London (Academic Press) Vol. 9, p. 69.
LATNER, A. L., GARDNER, P. S., TURNER, D. M. AND BROWN, J. O.—(1964) *Lancet*, i, 197.
LATNER, A. L. AND SKILLEN, A. W.—(1961) *Lancet*, ii, 1286.
LATNER, A. L. AND TURNER, D. M.—(1967) *Clinica chim. Acta*, **15**, 97.
MAHY, B. W. J., ROWSON, K. E. K. AND SALAMAN, M. H.—(1964) *Virology*, **23**, 528.
MIRAND, E. A., STEEVES, R. A., AVILA, L. AND GRACE, J. T., Jr.—(1968) *Proc. Soc. exp. Biol. Med.*, **123**, 900.
NATELSON, S.—(1961) ' Microtechniques of Clinical Chemistry '. Springfield, Illinois (C. C. Thomas) p. 309.
NOTKINS, A. L.—(1965) *Bact. Rev.*, **29**, 143.
NOTKINS, A. L., BERRY, R. J., MOLONEY, J. B. AND GREENFIELD, R. E.—(1962) *Nature, Lond.*, **193**, 79.
PFLEIDERER, G. AND WACHSMUTH, E. D.—(1961) *Klin. Wschr.*, **39**, 352.
PLAGEMANN, P. G. W., GREGORY, K. F., SWIM, H. E. AND CHAN, K. K. W.—(1963) *Can. J. Microbiol.*, **9**, 75.
PLAGEMANN, P. G. W., WATANABE, M. AND SWIM, H. E.—(1962) *Proc. Soc. exp. Biol. Med.*, **111**, 749.
RILEY, V.—(1963*a*) *N.Y. St. J. Med.*, **63**, 1522.—(1963*b*) *Ann. N.Y. Acad. Sci.*, **100**, 762.
RILEY, V., LILLY, F., HUERTO, E. AND BARDELL, D.—(1960) *Science, N.Y.*, **132**, 545.
SAITO, T., OHIRA, S. AND KANAMARU, R.—(1968) *Tohoku J. exp. Med.*, **96**, 63.
SMITHIES, O.—(1955) *Biochem. J.*, **61**, 629.
THIERY, M. AND WILLIGHAGEN, R. G. J.—(1965) *Lancet*, ii, 244.
VAN VALS, G. H., BOSCH, L. AND EMMELOT, P.—(1956) *Br. J. Cancer*, **10**, 792.
VESELL, E. S.—(1963) in ' Protides of the Biological Fluids ', edited by Peeters, H. Amsterdam (Elsevier) p. 150.
WARNOCK, M. L.—(1964) *Proc. Soc. exp. Biol. Med.*, **115**, 448.
WENNER, C. E., MILLIAN, S. J., MIRAND, E. A. AND GRACE, J. T., Jr.—(1962) *Virology*, **18**, 486.


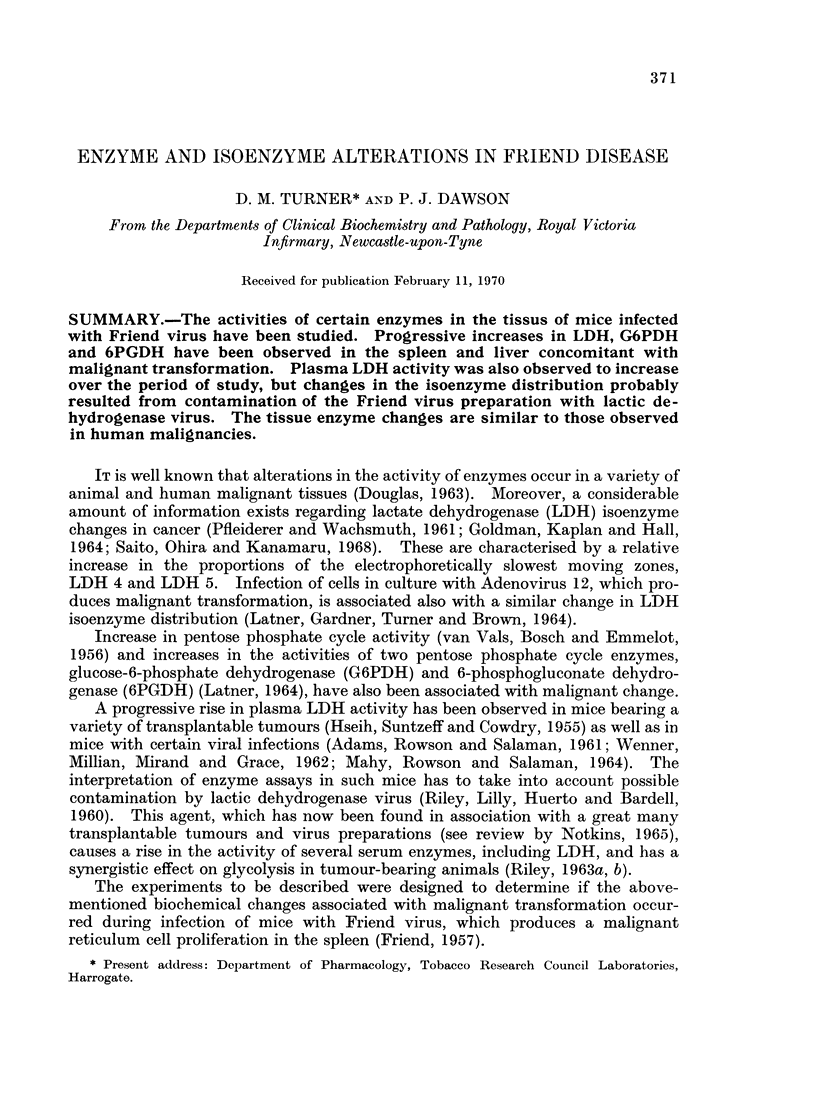

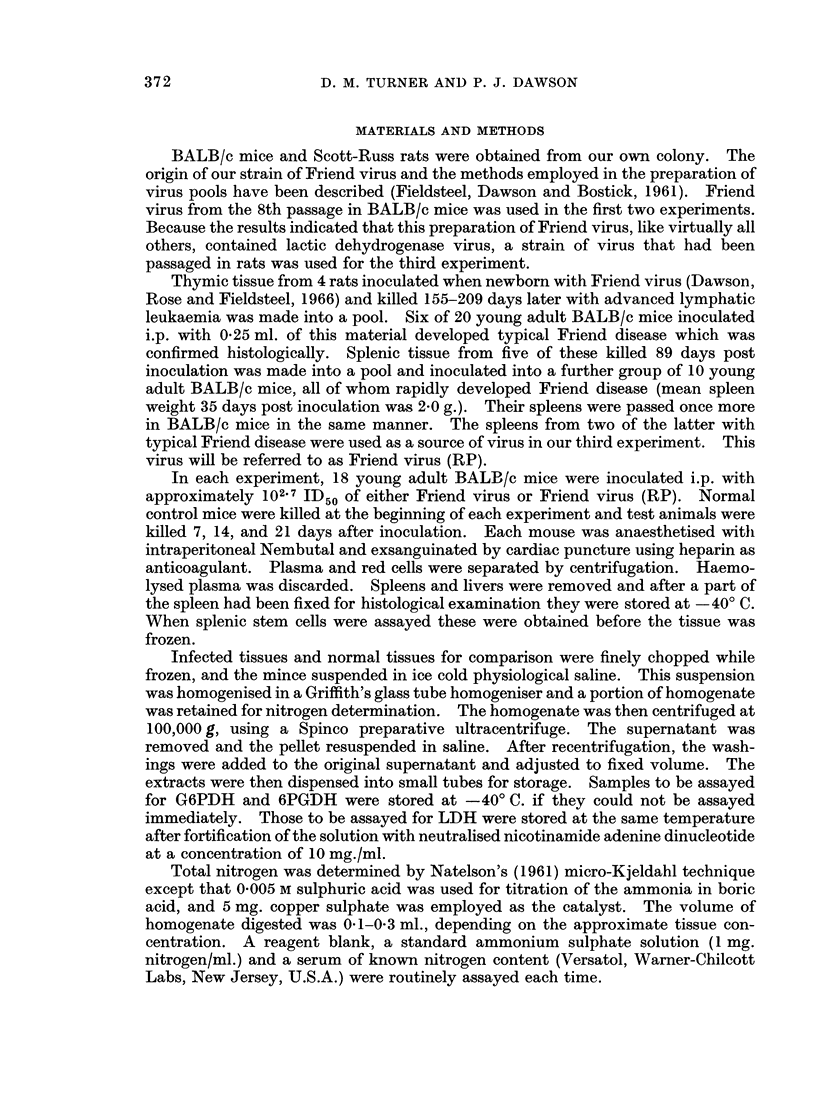

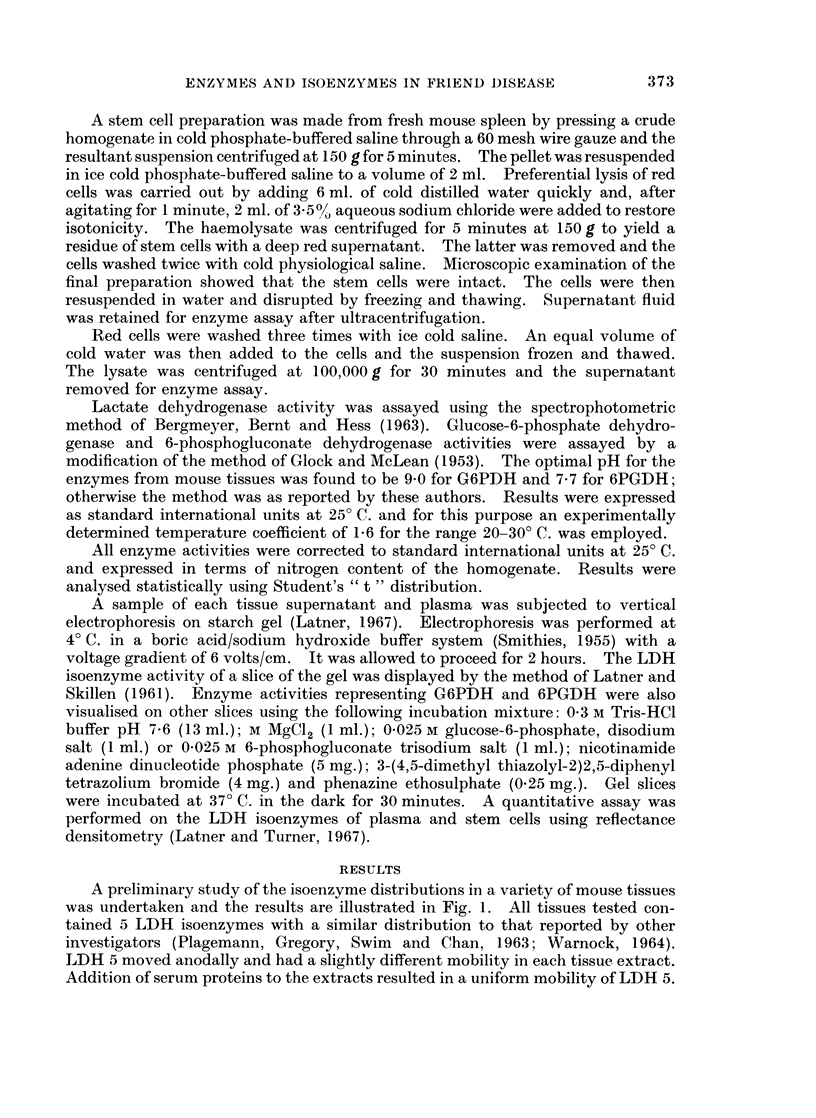

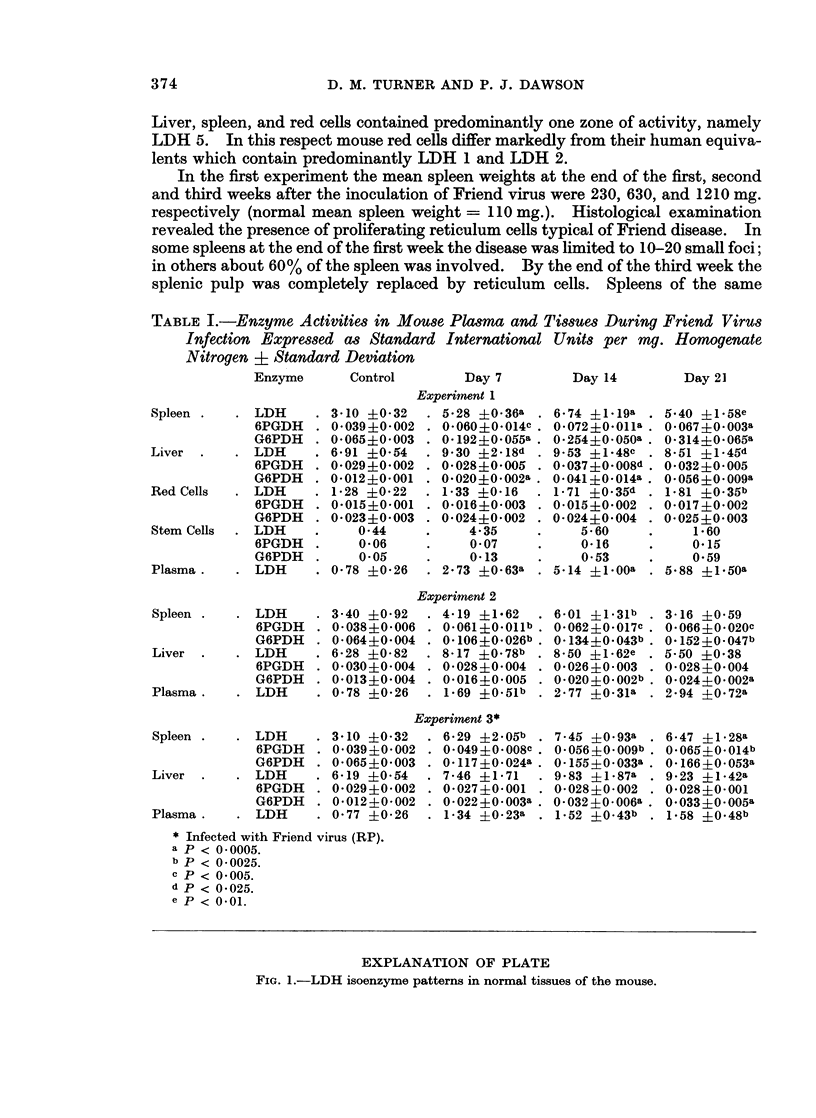

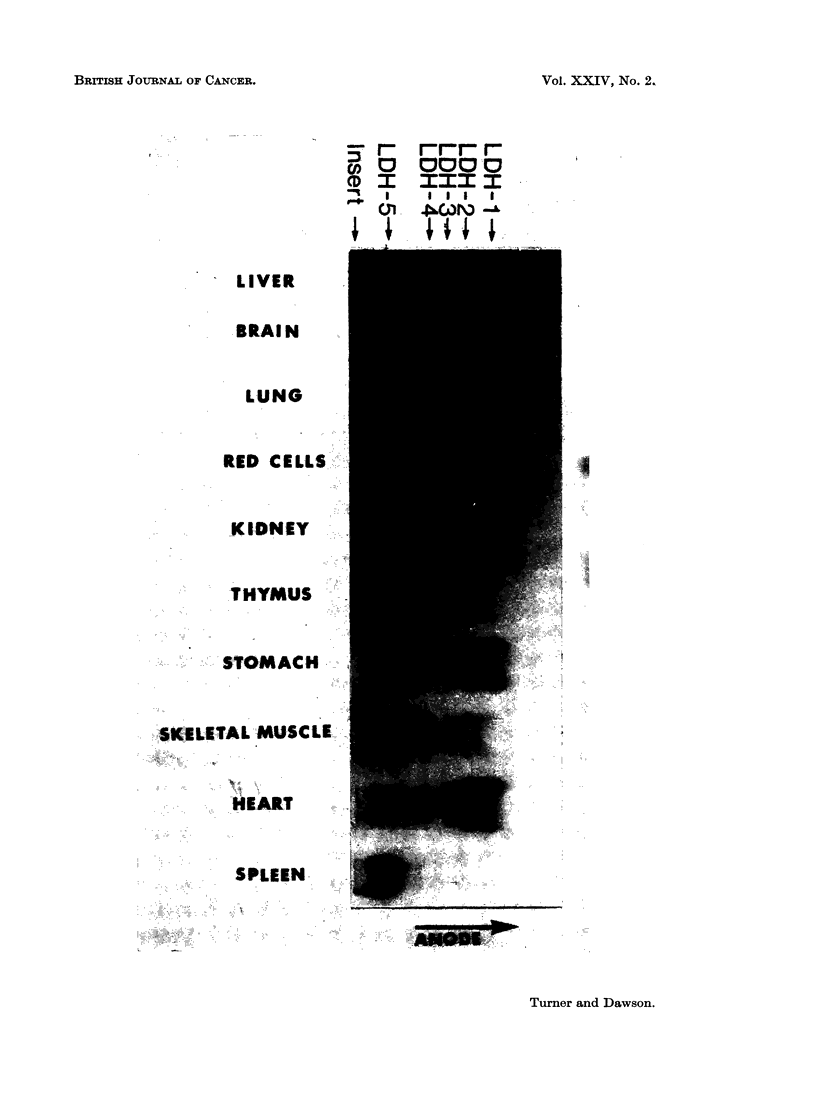

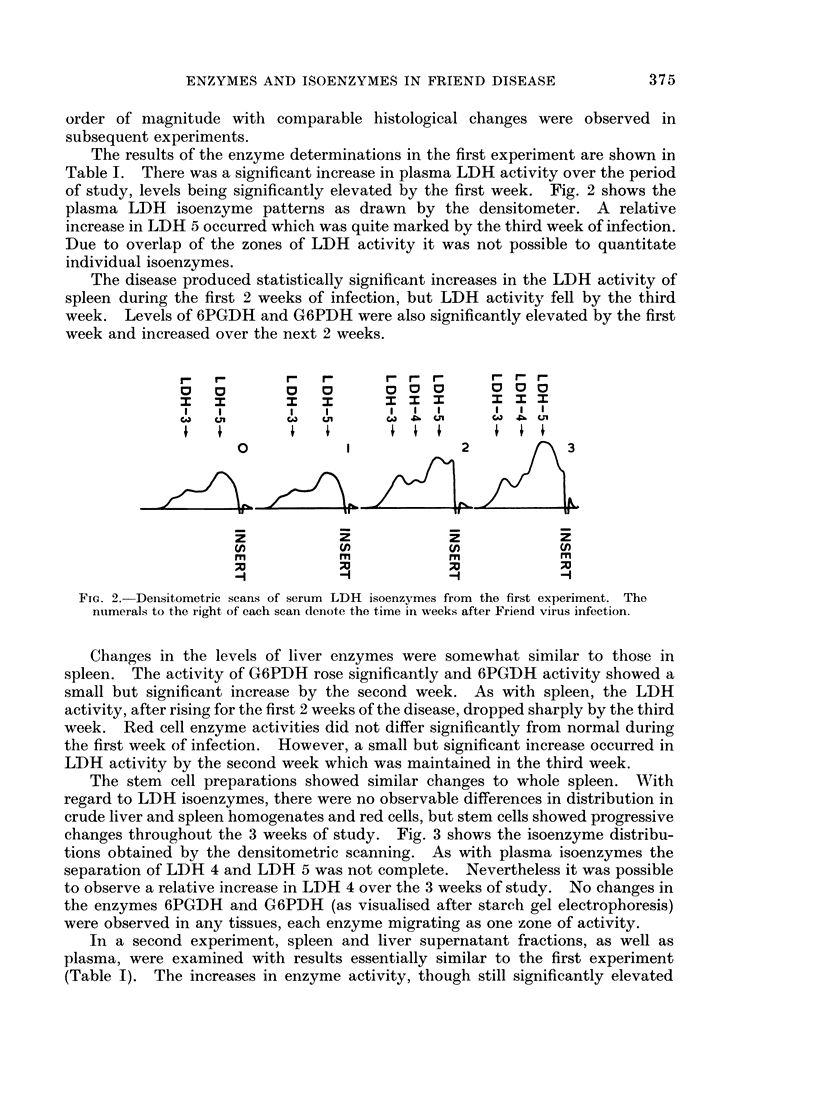

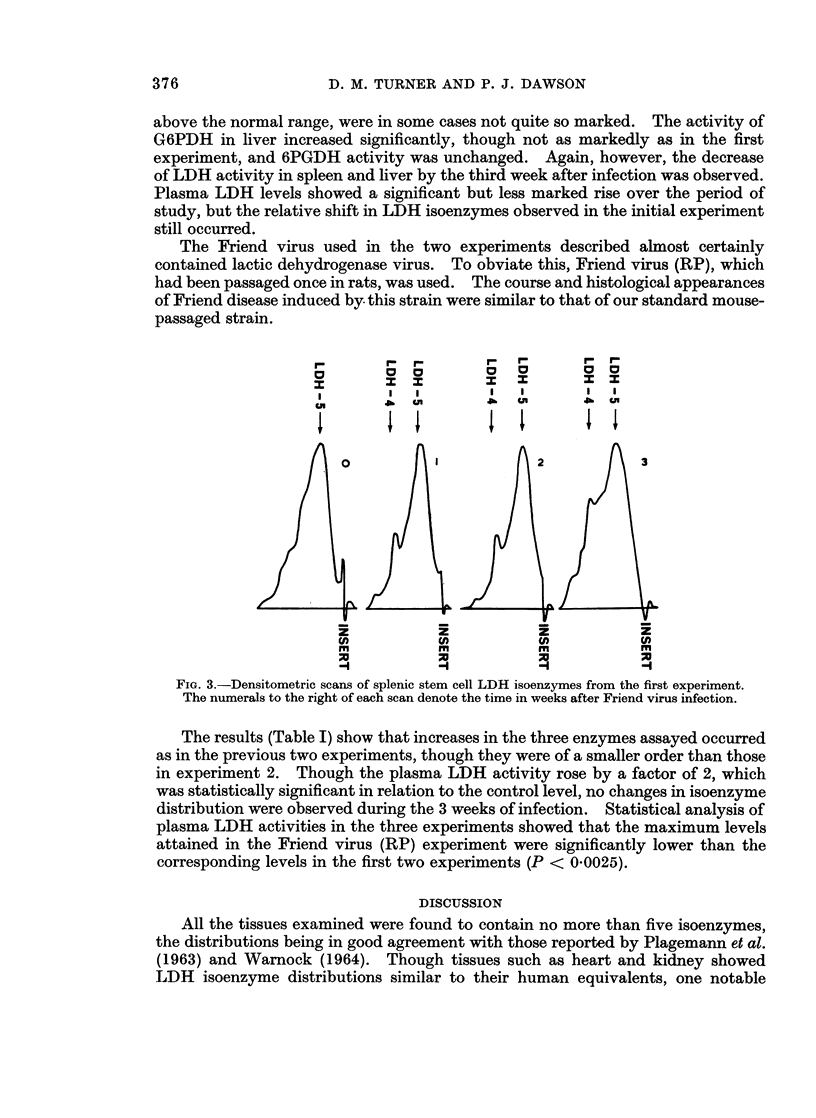

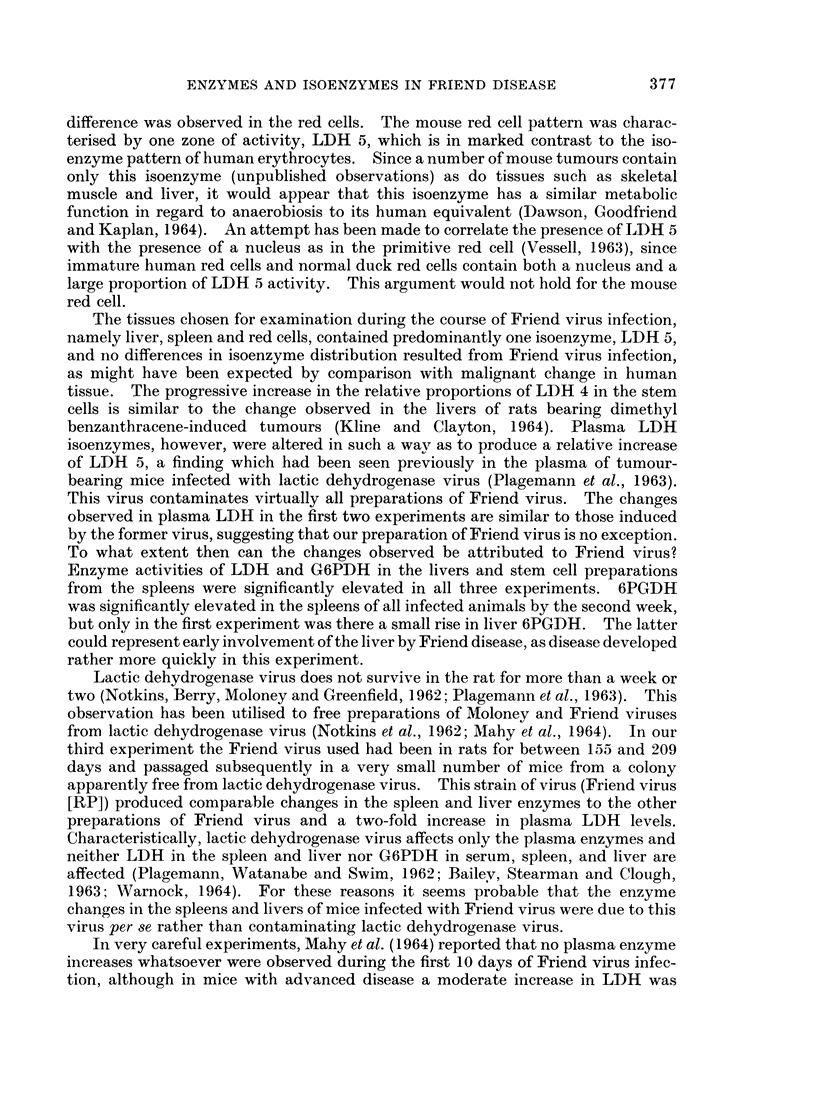

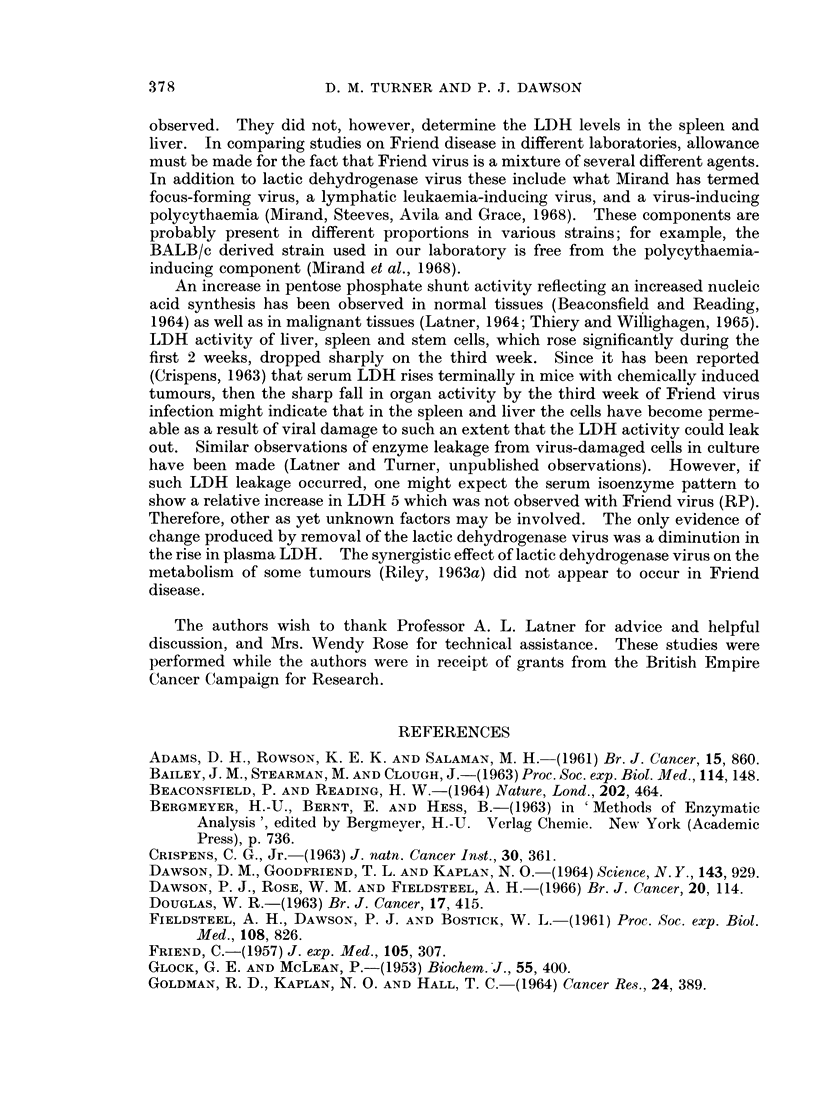

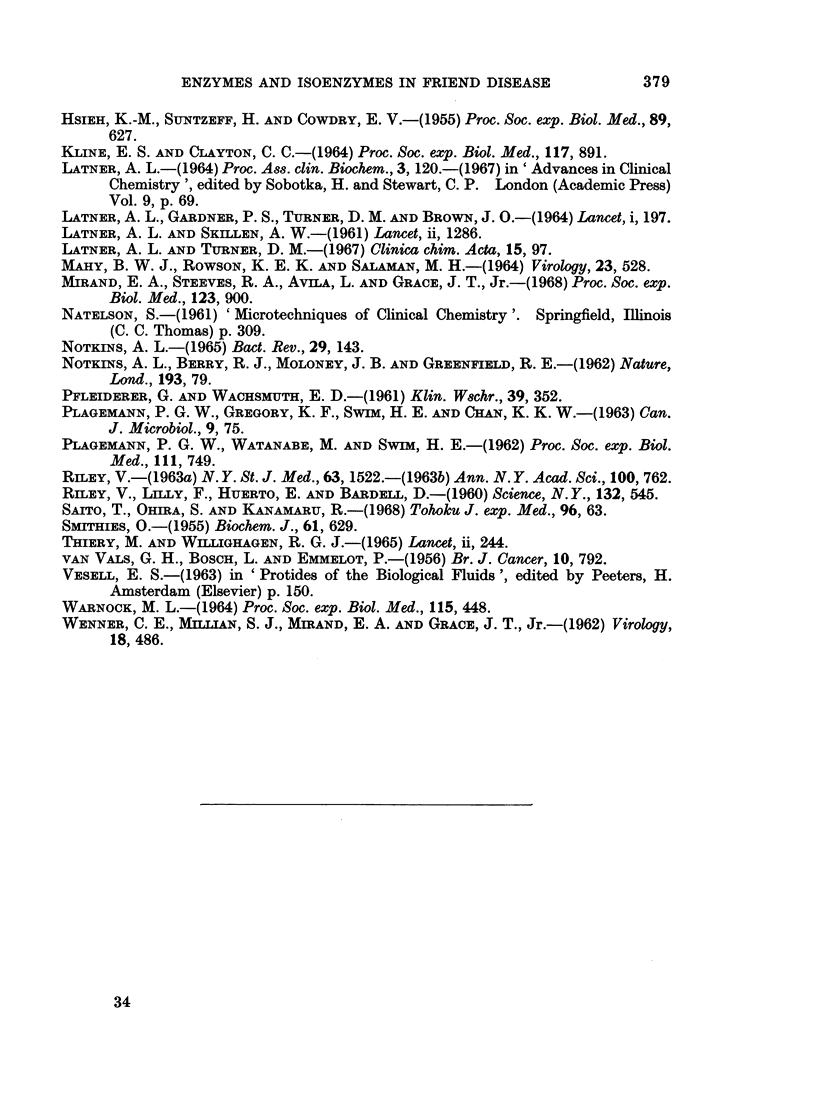


## References

[OCR_00638] ADAMS D. H., ROWSON K. E., SALAMAN M. H. (1961). The effect of tumours, of leukaemia, and of some viruses associated with them, on the plasma lactic dehydrogenase activity of mice.. Br J Cancer.

[OCR_00639] BAILEY J. M., STEARMAN M., CLOUGH J. (1963). LDH LEVELS IN BLOOD AND TISSUES OF MICE INFECTED WITH AN LDH AGENT.. Proc Soc Exp Biol Med.

[OCR_00640] BEACONSFIELD P., READING H. W. (1964). PATHWAYS OF GLUCOSE METABOLISM AND NUCLEIC ACID SYNTHESIS.. Nature.

[OCR_00647] CRISPENS C. G. (1963). Serum lactic dehdrogenase levels in mice during the development of autochthonous and chemically induced tumors.. J Natl Cancer Inst.

[OCR_00649] DAWSON D. M., GOODFRIEND T. L., KAPLAN N. O. (1964). LACTIC DEHYDROGENASES: FUNCTIONS OF THE TWO TYPES RATES OF SYNTHESIS OF THE TWO MAJOR FORMS CAN BE CORRELATED WITH METABOLIC DIFFERENTIATION.. Science.

[OCR_00650] Dawson P. J., Rose W. M., Fieldsteel A. H. (1966). Lymphatic leukaemia in rats and mice inoculated with Friend virus.. Br J Cancer.

[OCR_00653] FIELDSTEEL A. H., DAWSON P. J., BOSTICK W. L. (1961). Quantitative aspects of Friend leukemia virus in various murine hosts.. Proc Soc Exp Biol Med.

[OCR_00657] FRIEND C. (1957). Cell-free transmission in adult Swiss mice of a disease having the character of a leukemia.. J Exp Med.

[OCR_00659] GLOCK G. E., McLEAN P. (1953). Further studies on the properties and assay of glucose 6-phosphate dehydrogenase and 6-phosphogluconate dehydrogenase of rat liver.. Biochem J.

[OCR_00661] GOLDMAN R. D., KAPLAN N. O., HALL T. C. (1964). LACTIC DEHYDROGENASE IN HUMAN NEOPLASTIC TISSUES.. Cancer Res.

[OCR_00669] KLINE E. S., CLAYTON C. C. (1964). LACTIC DEHYDROGENASE ISOZYMES DURING DEVELOPMENT OF AZO DYE TUMORS.. Proc Soc Exp Biol Med.

[OCR_00677] LATNER A. L., GARDNER P. S., TURNER D. M., BROWN J. O. (1964). EFFECT OF A POSSIBLE ONCOGENIC VIRUS (ADENOVIRUS TYPE 12) ON LACTATE DEHYDROGENASE IN TISSUE CULTURE.. Lancet.

[OCR_00681] MAHY B. W., ROWSON K. E., SALAMAN M. H. (1964). PLASMA ENZYME LEVELS IN VIRUS-INFECTED MICE.. Virology.

[OCR_00683] Mirand E. A., Steeves R. A., Avila L., Grace J. T. (1968). Spleen focus formation by polycythemic strains of Friend leukemia virus.. Proc Soc Exp Biol Med.

[OCR_00691] NOTKINS A. L. (1965). LACTIC DEHYDROGENASE VIRUS.. Bacteriol Rev.

[OCR_00699] PFLEIDERER G., WACHSMUTH E. D. (1961). [The heterogenicity of lactic dehydrogenase in the ontogeny and pathology of man].. Klin Wochenschr.

[OCR_00705] PLAGEMANN P. G., WATANABE M., SWIM H. E. (1962). Plasma lactic dehydrogenase-elevating agent of mice: effect on levels of additional enzymes.. Proc Soc Exp Biol Med.

[OCR_00708] Riley V., Lilly F., Huerto E., Bardell D. (1960). Transmissible Agent Associated with 26 Types of Experimental Mouse Neoplasms.. Science.

[OCR_00710] SMITHIES O. (1955). Zone electrophoresis in starch gels: group variations in the serum proteins of normal human adults.. Biochem J.

[OCR_00714] VAN VALS G. H., BOSCH L., EMMELOT P. (1956). The metabolism of neoplastic tissues: carbon dioxide production from specifically 14C-labelled glucose by normal and neoplastic tissues.. Br J Cancer.

[OCR_00720] WARNOCK M. L. (1964). ISOZYMIC PATTERNS IN ORGANS OF MICE INFECTED WITH LDH AGENT.. Proc Soc Exp Biol Med.

[OCR_00722] WENNER C. E., MILLIAN S. J., MIRAND E. A., GRACE J. T. (1962). Serum lactic dehydrogenase levels of mice inoculated with oncogenic and non-oncogenic viruses.. Virology.

